# Ameliorative effects and mechanisms of paeonol against rosacea through network pharmacology and experimental validation

**DOI:** 10.3389/fimmu.2026.1949398

**Published:** 2026-09-28

**Authors:** Manyu Chen, Yueye Xu, Xinyu Li, Jingang Xu, Boya Xu, Tingting Zhang, Yujun Shuai, Yao Zhang, Qingquan Chang, Xiaoniu Tang, Jinhong Zhao, Yuanyuan Li

**Affiliations:** 1 Department of Medical Parasitology, Wannan Medical University, Wuhu, China; 2 Department of Basic Medical Sciences, Clinical College of Anhui Medical University, Hefei, China; 3 School of Public Health, Wannan Medical University, Wuhu, China; 4 School of Basic Medical Sciences, Wannan Medical University, Wuhu, China; 5 School of Clinical Medicine, Wannan Medical University, Wuhu, China; 6 Anhui Provincial Key Laboratory of Biological Macro-molecules, Wannan Medical University, Wuhu, China

**Keywords:** LL-37, NF-κB, paeonol, rosacea, S100A9

## Abstract

Rosacea is a long-term and relapsing inflammatory skin disorder resulting from dysfunction of the immune and/or neurovascular systems. Paeonol (Pae), the primary active component extracted from Moutan Cortex, exhibits various pharmacological effects, including anti-inflammatory and antioxidant properties. This study aimed to evaluate the therapeutic efficacy of Paeonol in treating rosacea and to investigate the underlying mechanisms of its action. Bioinformatics analysis was employed to assess the overlap between rosacea pathogenesis and the pharmacological targets of Pae. An LL-37-induced rosacea-like mouse model was established to evaluate the therapeutic effects of Pae *in vivo*. Histopathological analysis was performed to assess skin lesions, angiogenesis, immune cell infiltration, and cytokine production. The involvement of the S100A9-NF-κB pathway was investigated to elucidate the molecular mechanism. Bioinformatics analysis predicted potential overlapping molecular targets between rosacea pathogenesis and paeonol.*In vivo*, Pae intervention markedly ameliorated rosacea-like skin lesions. Mechanistically, Pae suppressed the production of S100A9 through modulation of the LL-37 and NF-κB pathways. This study indicates that paeonol mitigates skin lesions in LL-37-induced rosacea-like mice, and these therapeutic effects are correlated with the modulation of the S100A9-NF-κB signaling axis, suggesting its potential as a promising candidate agent for rosacea-like inflammation intervention.

## 1 Introduction

Rosacea is a chronic and recurrent facial inflammatory skin disorder that affects people throughout the world. Epidemiological data have shown that the incidence of rosacea is greater than 5% globally (1–3). It mainly affects the central skin of the face and is characterized by relapsing flushing, sustained erythema, papules, telangiectasias, phymatous changes, edema, and pustules, occurring alone or in combination (4, 5). Clinical presentations have revealed that rosacea mainly involves erythematotelangiectatic (ETR), phymatous (PR), and ocular and papulopustular (PPR) subtypes, which may not be independent of each other (6–8). Although the pathophysiological mechanisms of rosacea are ambiguous, many studies have reported that immune and/or neurovascular dysfunction are major contributors to the pathogenesis of rosacea and impair the skin barrier (4, 9, 10). Patients with rosacea have several flare triggers, including temperature changes, exposure to ultraviolet radiation, exercise, stress, diet, and alcohol. These factors make patients more vulnerable to skin disorders because they aggravate the innate immune response and/or neurovascular dysfunction (4, 11, 12). Rosacea may impose physical and mental burdens even if it does not directly threaten the lives of patients. Rosacea can impose additional economic burdens on patients because of its high recurrence rate and ineffective treatment, as the molecular mechanisms underlying its pathogenesis are unclear (13–15).

Rosacea can be treated using different types of therapeutic interventions. Generally, retinoids and oral or topical antibiotics are the most widely used pharmacological treatments. However, the long-term application of oral or topical antibiotics may lead to antibiotic resistance, which can increase the incidence of rosacea (16). Administering isotretinoin orally may affect the development of fetal organs and cause headaches and dry skin; its repeated long-term use can also lead to osteoporosis (13, 17). Thus, novel therapeutic techniques need to be developed to treat rosacea.

Paeonol (Pae), also called 2’-hydroxy-4’-methoxyacetophenone, is the major natural bioactive component isolated from Moutan Cortex root bark (Paeonia suffruticosa Andrew) (18–20). In modern China, Pae has long been used in clinical practice. Additionally, Pae injection has been adopted as a common treatment intervention in clinical settings since the 1970s (21, 22). Pae has various biological effects, such as anti-inflammatory, antioxidant, antitumor, antifungal, and insecticidal effects (19, 23–26). The NMPA has authorized Pae in China for treating inflammation and pain-related illnesses (27). Moreover, Pae has satisfactory therapeutic effects on pigmentation, psoriasis, eczema, atopic dermatitis, and itchy skin. Pae tablets and Pae ointment have been developed for clinical application (20, 28–30). However, a research gap exists regarding the efficacy of Pae in the alleviation of rosacea.

Cathelicidin (LL-37) is an antimicrobial compound with multifunctional properties in mammals (31). In humans, LL-37 is a unique cathelicidin-derived AMP (Antimicrobial Peptide). The 37-residue mature form of the AMP of hCAP18 was termed LL-37, based on the first two Leu residues (32). LL-37 has antimicrobial, immunomodulatory, antiviral, anticancer, anti-inflammatory, angiogenic, wound healing, and chemotactic activities (32, 33). In another study, a mouse model was used to investigate skin inflammation in LL-37-induced rosacea, which presented characteristics similar to those of rosacea patients. Therefore, researchers create animal models for rosacea research by administering LL-37 intradermally (34).

The protein S100A9, which belongs to the S100 protein family, is also called myeloid-related protein (MRP)-14. It is related to infection, inflammation, calcium signaling, neurological diseases in the brain, immune-related diseases, and the development of different types of cancer (35, 36). Recent studies have suggested that S100A9 aggravates inflammation in rosacea through the TLR4/MyD88/NF-κB pathway (37). Moreover, some researchers conducted skin transcriptome analysis and found that S100A9 worsens rosacea-related inflammation (38). Additionally, when the NF-κB pathway is abnormally activated, several inflammatory factors are produced and released, inducing chronic inflammation (39–41). As Pae is a drug with primary anti-inflammatory efficacy, investigating whether Pae mitigates LL-37-mediated rosacea through the S100A9-NF-κB signaling pathway can provide novel insights.

In this study, we investigated the medicinal properties of rosacea and its associated mechanisms based on network pharmacology and animal experiments. We aimed to explore potential therapeutic clues for rosacea treatment.

## 2 Materials and methods

### 2.1 Regents

Paeonol (H811202) and doxycycline hyclate (D832390) were purchased from Shanghai Macklin Biochemical Technology Co., Ltd. (Shanghai, China). LL-37 (HY-108331) was purchased from MedChemExpress (NJ, USA).

### 2.2 Screening of Pae-related targets

The SMILES, CID, and 2D/3D structure of Pae were obtained from PubChem (https://pubchem.ncbi.nlm.nih.gov). The candidate Pae targets were subsequently identified via TCMSP (https://www.tcmsp-e.com/#/home), HIT (http://www.badd-cao.net:2345/), BATMAN 2.0 (http://bionet.ncpsb.org.cn/batman-tcm/; score cutoff > 0.84), and Swiss Target Prediction (http://www.swisstargetprediction.ch/; probability>0). Finally, the data from the abovementioned databases were summarized, and the final Pae-related targets were obtained after removing duplicates.

### 2.3 Screening of rosacea-related genes

Setting “rosacea” as the keyword, the potential targets associated with rosacea, coded as GSE65914, were obtained from GEO (https://www.ncbi.nlm.nih.gov/geo; LogFCfilter = 1, adjPfilter = 0.05). The GSE65914 dataset comprises 58 samples, including 10 healthy volunteers and 19 rosacea patients, of whom 7 were diagnosed with ETR, 6 with PPR, and 6 with PhR based on the classification system of the American Rosacea Society (13).

### 2.4 Establishment of a protein-protein interaction network for intersecting targets of Pae with rosacea

The intersection target dataset of Pae and rosacea was obtained using the VennDiagram R package in R 4.3.3. Subsequently, the PPI network of the intersected targets of Pae with rosacea was established by inputting the intersected target dataset of paeonol and rosacea into the STRING database (https://string-db.org/), with the species specified as “Homo sapiens” and the minimum required interaction score > 0.4. After constructing the PPI network, the Cytoscape 3.8.2 software was used to identify and visualize the hub genes. Hub genes were screened according to degree centrality values calculated within Cytoscape; nodes with higher degree values were regarded as core hub genes.

### 2.5 Gene ontology and Kyoto Encyclopedia of Genes and Genomes analyses

The GO and KEGG analyses were performed using the R packages “org.Hs.eg.db”, “colorspace”, “stringi”, “DOSE”, “clusterProfiler”, “pathview”, “limma”, and “ggplot2”. Both the “p” and “q” values were set at 0.05. The GO terms were divided into three major classes: BPs, CCs, and MFs. The functions and biological implications of potential targets were identified by KEGG.

### 2.6 Verification via molecular docking analysis

For protein targets, their 3D structures were obtained from the RCSB database (https://www.rcsb.org/), repaired, and preconditioned (by removing metal ions and water molecules) via mgltools_win32_1.5.6. The 3D structure of Pae was obtained from the PubChem database (https://pubchem.ncbi.nlm.nih.gov/) and later optimized using the SYBYL-X 2.0 software under the following optimized parameters: triple force field, gas-huckel charge, maximum iteration coefficient of 10,000, energy gradient of 0.005 kcal/(mol·A), and other parameters with default values. Finally, it was molecularly docked with protein targets, and the docking results were visually presented to verify the interaction between Pae and the target proteins using AutoDock Vina 1.1.2 and Discovery Studio 2019 software.

### 2.7 Animal experiments

42 female, specific pathogen-free (SPF) BALB/c mice (age: 6-7 weeks old; weight: 20 ± 0.4 g; certificate no: SYXK [Anhui] 2023-005v) were obtained from Henan SCBS Biotechnology Co., Ltd. (Henan, China). Before the experiments, all mice were acclimated to the experimental protocols for two weeks and were housed under SPF conditions. Rosacea-like skin lesions were induced with 40 μL of LL-37 (320 μM), following the methods described in another study (1). 100 mg of paeonol was completely dissolved in 100 μL of DMSO to prepare the stock solution. Then, 10 μL, 20 μL, and 30 μL aliquots of the paeonol stock solution were separately withdrawn and further diluted to 1000 μL with PBS buffer, yielding working solutions at paeonol concentrations of 10 mg/mL, 20 mg/mL, and 30 mg/mL, respectively. These working solutions were administered via intraperitoneal injection. The corresponding doses in mice were 50 mg/kg/d, 100 mg/kg/d and 150 mg/kg/d, determined based on our preliminary experimental results. All animals were randomized into the control group (phosphate-buffered saline), rosacea model group (LL-37), doxycycline hyclate (DH) group (LL-37 + DH 30 mg/kg), and Pae groups (LL-37 + Pae 50, 100, and 150 mg/kg) (n = 7 mice/group). The detailed treatments for each mouse group are illustrated in **Figure 1a**. The severity of skin inflammation was assessed based on the erythema score and redness area following previously described methods (42), 12 h after administering the final injection. Next, the dorsal skin lesions of the mice were collected for protein isolation, RNA extraction, H&E staining, TB staining, and immunofluorescence analysis. Sample size selection was based on power analysis and previous similar research, ensuring adequate statistical power to identify treatment differences at a = 0.05 and 80% power. All animal procedures were approved by the Animal Ethics Committee of Wannan Medical College (Approval No. SUCMDL20220805001). The studies were conducted in accordance with the local legislation and institutional requirements.

**Figure 1:**
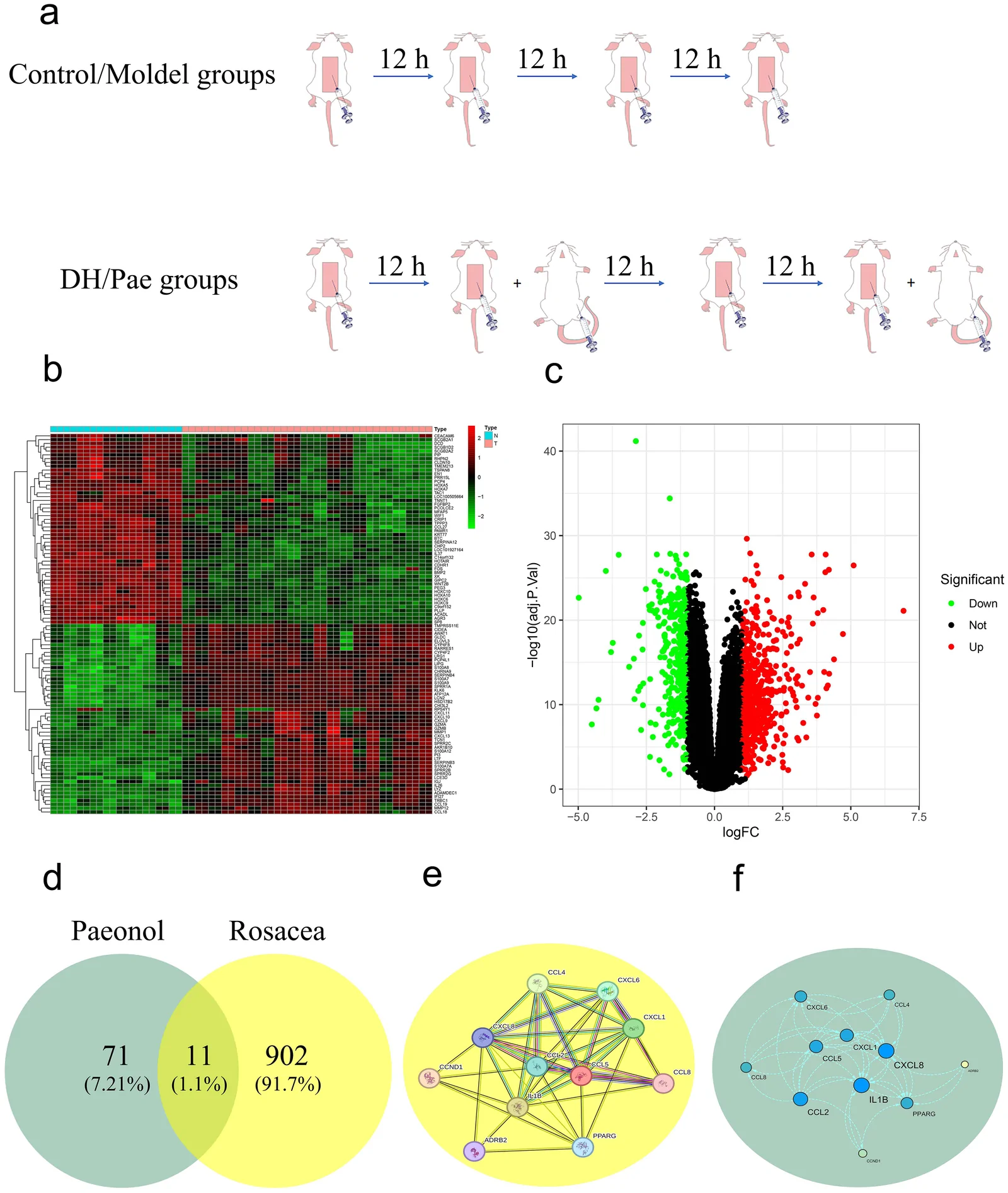
The interaction of mice and the network pharmacology of Pae and rosacea. **(a)** All groups were shaved 24 h before treatment. The model groups were injected intradermally on the back skin with LL-37 (40 μL, 320 μM) four times at intervals of 12 h. The control groups were also administered intradermal injections of PBS (40 μL) at the same frequency as the model groups. The DH and Pae groups received the first intradermal injection of an equivalent volume of LL-37 (320 μM). After the first interval of 12 h, the first injection was repeated in the DH and Pae groups, and the first intraperitoneal injection was performed at 30 mg/kg for DH and 50, 100, and 150 mg/kg for Pae. The DH and Pae groups were administered a third intradermal injection of LL-37 after the second interval of 12 h. At 12 h, the DH and Pae groups received the final intradermal and intraperitoneal injections at the same dose as the first intradermal and intraperitoneal injections. Then, 12 h after the last injection, all mice were sacrificed. **(b)** The heatmap shows the differentially expressed genes in rosacea. Red indicates upregulated differentially expressed genes, and green indicates downregulated differentially expressed genes. **(c)** The volcano plot illustrates differentially expressed genes. **(d)** The Venn diagram illustrates Pae-related targets and rosacea-related targets. **(e)** PPI network for overlapping proteins between Pae-related targets and rosacea-related targets. **(f)** Simplified PPI network for the overlapping proteins (larger circles and brighter colors indicate closer interactions).

### 2.8 Evaluation of skin inflammation severity

Twelve hours after the final injection, dorsal skin lesions were photographed, and rosacea-like severity was scored based on erythema intensity and area (42). Erythema severity was graded from 0 to 4: 0, no erythema; 1, faint erythema; 2, mild erythema; 3, obvious and well-defined erythema; 4, dark erythema with clear borders.

### 2.9 Hematoxylin and eosin staining

The dorsal skin samples of the mice were harvested. The samples were immersed in 4% paraformaldehyde, embedded in paraffin, and sliced into thin sections. These sections were subjected to H&E staining and observed under an optical microscope (Olympus, Tokyo, Japan).

### 2.10 Toluidine blue staining

Embedded tissue samples were cut into thin sections for TB staining following the affiliated instructions. The sections were examined using a light microscope (Olympus, Tokyo, Japan). Five fields of view were randomly selected under the microscope (200×), and mast cells in every field were counted.

### 2.11 Immunofluorescence staining

Following deparaffinization and rehydration, the paraffin sections were subjected to high-pressure repair and incubated with primary antibodies, which included S100A9 (1:200 dilution, Proteintech, Chicago, USA), CD4 (1:200 dilution, Bioss, Beijing, China), and CD31 (1:200 dilution, Affinity, Colorado, USA). Then, they were treated with 3% H_2_O_2_ at room temperature for 20 min. Next, the sections were incubated with goat anti-rabbit secondary antibodies (1:100 dilution, Affinity, Colorado, USA) for 20 min at 37 °C, followed by three washes with PBS. Finally, 4’,6-diamidino-2-phenylindole (DAPI) (Beyotime, Shanghai, China) was used to stain the sections. The expression of S100A9, CD4, and CD31 was monitored with a microscope (Olympus, Tokyo, Japan). Blue and green fluorescence indicated nuclei as well as S100A9, CD4, and CD31, respectively.

### 2.12 Enzyme-linked immunosorbent assay

We used ELISA kits for S100A9 (Rabbit mAb, Cat No.86211-1-PBS, ProteinTech), CCL2 (Rabbit mAb, Cat No. 83157-3-PBS, ProteinTech), and IL-1β (Rabbit mAb, Cat No. 84136-2-PBS, ProteinTech) (Ruixin Biotech, Quanzhou, China) to assess their contents in separate serum samples from each individual as recommended. All kits were based on quantitative sandwich enzyme immunoassay technology. Endotoxin was not present in any of the substances used.

### 2.13 Western blotting analysis

We used RIPA lysis buffer containing a protease/phosphatase inhibitor cocktail for extracting proteins from tissues. Then, the protein concentrations were measured with a BCA protein quantification kit (Monad, Suzhou, China). All samples were loaded onto 10% SDS-PAGE gels before being transferred onto PVDF membranes. After blocking with 5% nonfat milk at ambient temperature for 2 h, the membranes were washed with TBST, followed by overnight incubation with primary antibodies at 4 °C. After washing, they were probed with the corresponding HRP-labeled secondary antibodies (1:10000) at ambient temperature for 1 h, followed by enhanced chemiluminescence (ECL) detection. The following antibodies were used: phospho-NF-κB p65, NF-κB p65 (Cell Signaling Technology, Massachusetts, US), S100A9 (Abcam, Cambridge, UK), GAPDH (ABclonal, Wuhan, China), and goat anti-rabbit IgG (Biosharp, Hefei, China).

### 2.14 Quantitative real-time PCR

First, TRIzol reagent (ShareBio, Shanghai, China) was used to separate total RNA from the dorsal skin tissue of mice. The content and purity of RNA were determined using a NanoDrop spectrophotometer before conducting further analyses. RNA samples (2 μg) were reverse-transcribed into cDNA using an All-in-One First-Strand Synthesis MasterMix Kit with dsDNase (BestEnzymes, Lianyungang, China) as recommended. Then, the cDNA template was used for qPCR with Taq SYBR^®^ Green qPCR premix (BestEnzymes, Lianyungang, China) with a LightCycler 96 (Roche) thermocycler. The 2^–ΔΔCt^ approach was used for quantifying target gene levels, with GAPDH used as the reference. The primers were synthesized by Sangon Biotech (Shanghai, China). The sequences of primers used are shown in **Table 1**.

**Table 1:** The primer sequences used in RT-qPCR.

Target gene	Forward primers	Reverse primers
Mouse GAPDH	ACCACAGTCCATGCCATCAC	GTGAGGGAGATGCTCAGTGT
Mouse IL-1β	AAAAAAGCCTCGTGCTGTCG	GTCGTTGCTTGGTTCTCCTTG
Mouse CXCL8	TGTTCACAGGTGACTGCTCC	AGCCCATAGTGGAGTGGGAT
Mouse CCL2	CCACTCACCTGCTGCTACTCATTC	CTGCTGCTGGTGATCCTCTTGTAG
Mouse S100A9	CCACCATCATCGACACCTTCCATC	AGGTTGCCAACTGTGCTTCCAC

### 2.15 Statistical analysis

All statistical analyses were performed with GraphPad Prism 8.0 and SPSS 26.0. The values are presented as the mean ± standard error of the mean (SEM). The determination of the redness area and quantification of the IF and WB results were performed using the ImageJ software. The Shapiro–Wilk test was used to assess data normality. For normally distributed data, comparisons between two groups were made using Student’s t-test, and multiple groups were analyzed using one-way analysis of variance. For non-normally distributed data, the Kruska-Wallis test was used. Correlations between numerical variables were calculated using Pearson or Spearman correlation coefficients, depending on data distribution. Statistical significance was set at p < 0.05.

## 3 Results

### 3.1 The key blinding targets of Pae and rosacea are IL-1β, CXCL8, and CCL2

To identify the potential mechanisms and pathways by which Pae treats rosacea, we first screened Pae-related and rosacea-related targets. Setting “paeonol” as a keyword, we summarized the data obtained from the PubChem, TCMSP, BATMAN 2.0, HIT, and Swiss Target Prediction databases and identified 96 Pae action targets after removing duplicates. We identified 913 genes in the GEO database with the keyword “rosacea”, which included 552 upregulated genes and 361 downregulated genes (**Figures 1b, c**).

Next, we obtained 11 intersecting targets between paeonol-related targets and rosacea-related DEGs via Venn diagram analysis, including ADRB2, IL-1β, CCND1, CXCL6, CXCL1, CXCL8, CCL8, CCL5, CCL4, CCL2, and PPARG (**Figures 1d, e**). Subsequently, these 11 overlapping molecules were imported into the STRING database to construct a PPI network for protein-protein interaction visualization. This PPI network contained 11 nodes and 396 edges (**Figure 1d**). The top three key targets were CXCL8, IL-1β, and CCL2 (**Figure 1f**).

KEGG enrichment analyses and Gene Ontology (GO) facilitated the determination of the mechanism underlying the therapeutic effects of Pae on rosacea. GO annotations involving biological processes (BPs), cellular components (CCs), and molecular functions (MFs) revealed 1334 BPs, and the top five processes were leukocyte chemotaxis, cell chemotaxis, and humoral immune response. A total of 57 CCs were identified, and the top five cell components included the external side of the plasma membrane, collagen-containing extracellular matrix, cornified envelope, secretory granule membrane, and secretory granule lumen. A total of 133 MFs were screened, and the top five MFs were chemokine receptor binding, cytokine activity, chemokine activity, receptor ligand activity, and signaling receptor activator activity (**Figures 2a–d**). Overall, 1524 genes were included in the GO enrichment analysis (**Figure 2a**). Additionally, CCL2 was involved in the top five BPs, but IL-1β and CXCL8 participated in only the top four BPs. With respect to the top five CC terms, none of the top three key targets were enriched in the top five CC terms. Both CCL2 and CXCL8 participated in the top five MFs, but IL-1β was not involved in any of the top five MFs. KEGG analysis revealed 80 candidate pathways, with the five most closely associated pathways (**Figures 2e, f**), including viral protein interactions with cytokines and cytokine receptors, cytokine-cytokine receptor interactions, rheumatoid arthritis, the hematopoietic cell lineage, and the chemokine signaling pathway. However, CCL2 and CXCL8 were present in only four closely enriched pathways, including viral protein interactions with cytokines and cytokine receptors, cytokine-cytokine receptor interactions, the chemokine pathway, and rheumatoid arthritis. IL-1β functions only in rheumatoid arthritis and cytokine-cytokine receptor interactions.

**Figure 2:**
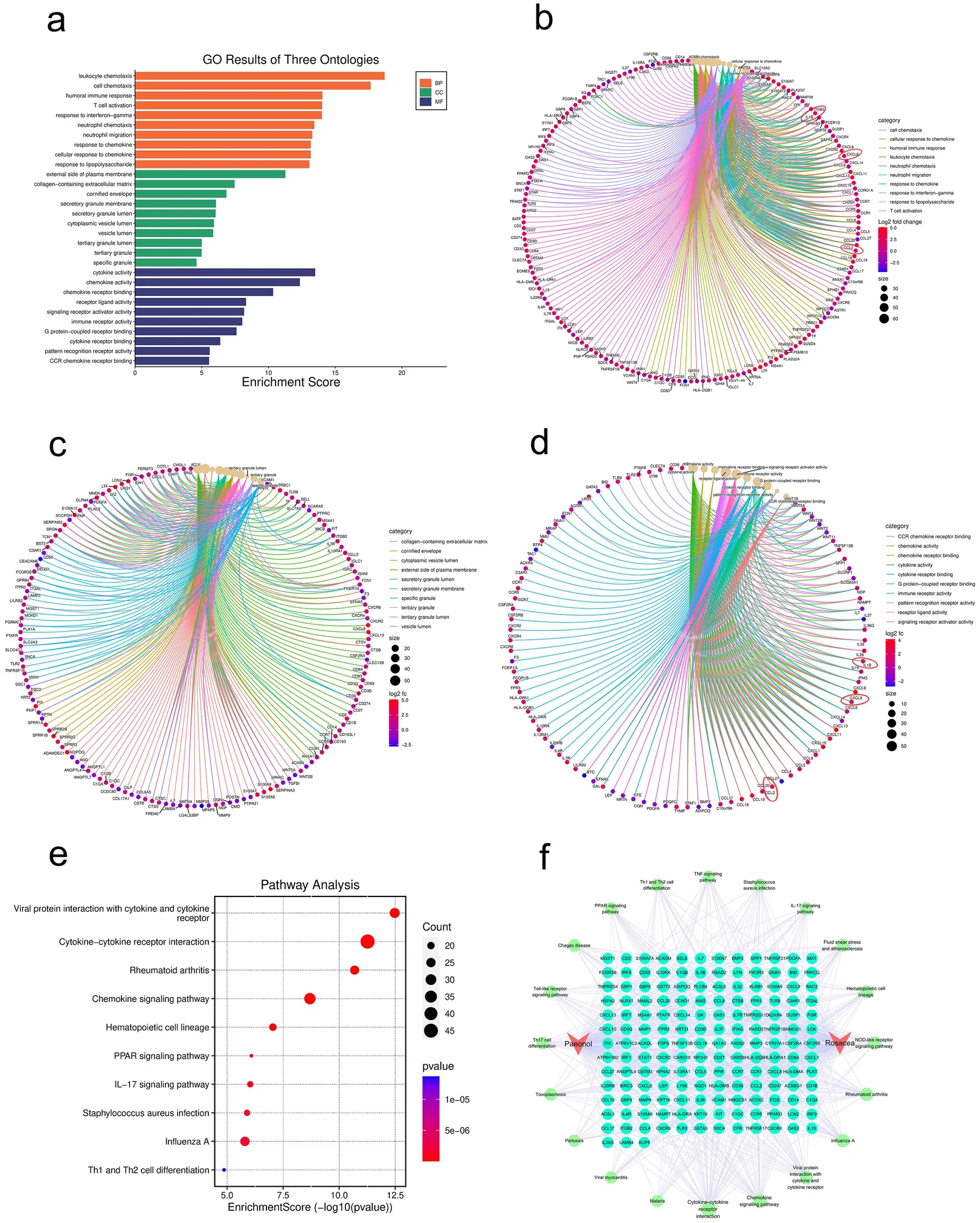
Enrichment analysis of Pae and rosacea. **(a)** Total GO annotation involving biological processes (BPs), cellular components (CCs), and molecular functions (MFs). GO annotation of **(b)** BPs, **(c)** CCs, and **(d)** MFs. **(e)** KEGG analysis of pharmacological target genes of Pae. **(f)** Network of targets and pathways associated with the anti-rosacea activity of Pae. The circles, diamonds, and circles from inside to outside represent the target, paeonol/rosacea, and pathway, respectively. Node size: greater nodes suggest greater importance.

We performed molecular docking analysis for core targets to provide theoretical structural clues regarding potential interactions between paeonol and candidate targets. Owing to the presence of three interacting targets of Pae and rosacea with too low an affinity to achieve molecular docking, we did not follow them further. Virtual verification of the remaining eight intersection targets of Pae and rosacea was performed using the AutoDock Vina software. This software was also used to calculate the best binding energies of Pae with eight target proteins, with lower values indicating greater binding ability. As shown in **Table 1**, 87.5% of the binding energies were < –5 kcal/mol, which suggests potential intermolecular interactions between paeonol and these candidate targets (43). The docking diagrams of Pae and three key targets are illustrated in **Figure 3a**. The main type of bonding observed was hydrogen bonding, which significantly increases the strength of binding between Pae and the target. Such modelled interactions may support the structural stability of the bonded structure, though functional relevance remains unproven experimentally. After modeling using PyMOL, Pae was predicted to interact with the amino acids GLN-38 and VAL-41 present in IL-1β, and the bond lengths were 2.4 Å and 2.3 Å, respectively. Additionally, the receptor–ligand binding free energy was predicted to be -5.1 kcal/mol (**Table 2**). Similarly, Pae was predicted to interact with the amino acids ASN-311 and PHE-312 found in CXCL8, and their bond lengths were 2.4 Å and 2.6 Å, respectively. The binding free energy was predicted to be -5.2 kcal/mol (**Table 2**). Pae was predicted to interact with ARG-8 and PHE-133 of CCL2, exhibiting lengths of 2.1, 2.7, and 2.5 Å, respectively. The binding free energy was predicted to be -5.0 kcal/mol (**Table 2**).

**Figure 3:**
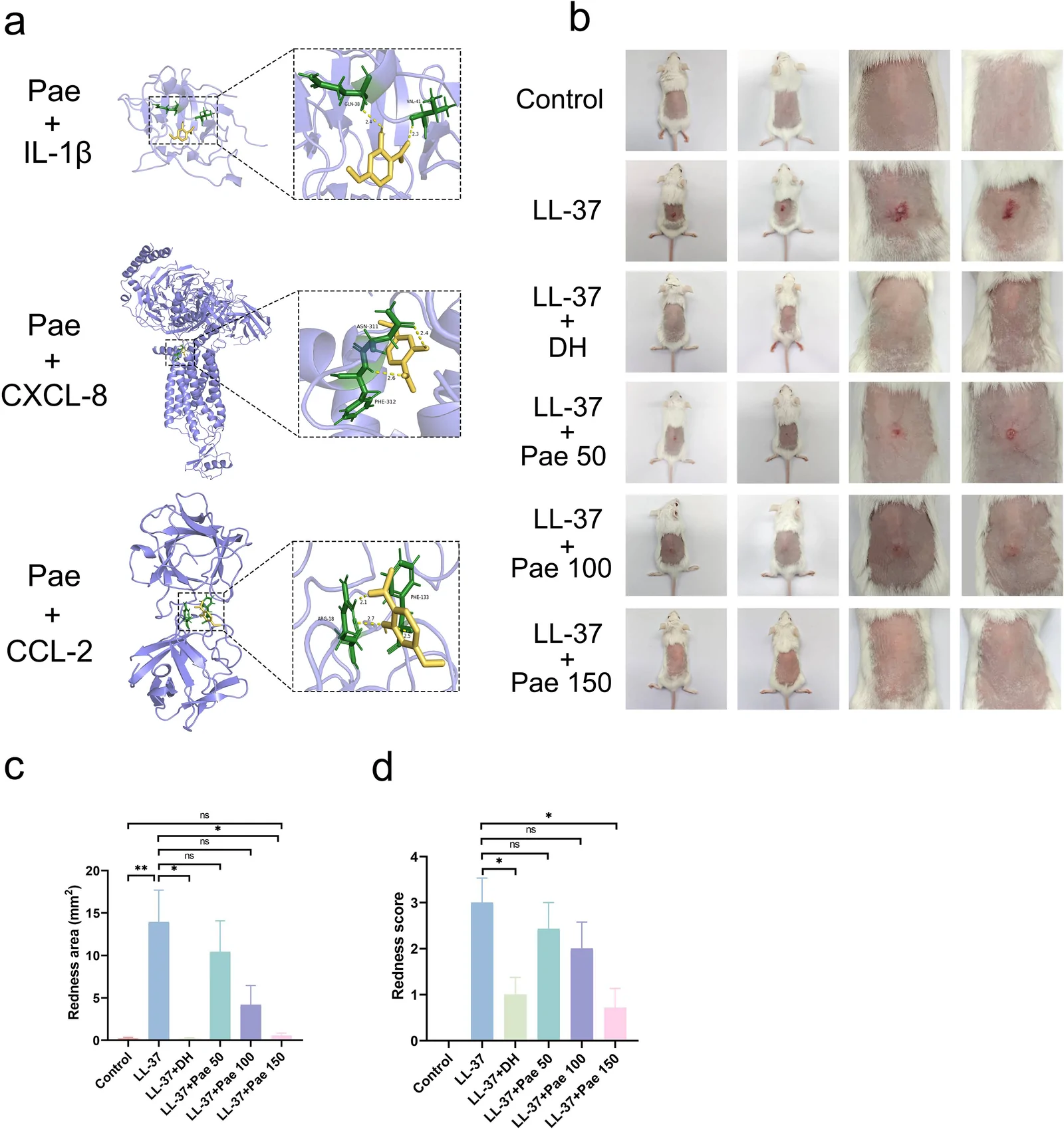
Virtual docking diagram for Pae with three key targets, and Pae suppressed the rosacea-like phenotype in LL-37-exposed mice. **(a)** Molecular docking results for Pae with IL-1β, CXCL8, and CCL2. **(b)** ALL skin condition images of all treated groups, with identical imaging conditions applied to all mice. **(c, d)** The severity of the rosacea-like phenotype was assessed according to the redness area and score (n = 7). The data are presented as the mean ± standard error; *p < 0.05 and **p < 0.01.

**Table 2:** The affinities between Pae and core targets.

	Target gene	PDB code	Affinity (kcal/mol)
Paeonol	ADRB2	3D4S	-6.5
	CCND1	2W99	-6.5
	PPARG	1PRG	-5.9
	CCL4	4MHE	-5.3
	CXCL8	8IC0	-5.2
	IL-1β	5R8Q	-5.1
	CCL2	4USP	-5.0
	CCL5	6STC	-4.9

These results suggest that Pae might be a therapeutic alternative for managing rosacea. Further investigations revealed that Pae exerts its primary effects through three key targets, including IL-1β, CXCL8, and CCL2. However, these results are virtual and require further confirmation through animal experimentation. Nevertheless, these hub targets were distributed across numerous enriched pathways. Given the frequent occurrence of S100A9 among these pathways and its documented pathogenic role in rosacea via NF-κB, we focused on the S100A9-NF-κB signaling axis for subsequent *in vivo* validation (38).

### 3.2 Pae can alleviate LL-37-induced rosacea-like skin inflammation in mice

To evaluate whether Pae can ameliorate LL-37-induced rosacea-like skin lesions in mice, we established a rosacea mouse model with LL-37, which is a well-recognized animal model. Pae was evaluated for its ability to resist inflammation in LL-37-mediated rosacea mice treated with Pae at different doses (50, 100, and 150 mg/kg) once daily for two consecutive days. At 12 h after the final injection was administered, we scored and photographed skin inflammation in each group of mice, as documented in previous studies. Compared to control mice, model mice presented noticeable erythema and telangiectasia and even pustules (**Figure 3b**), suggesting that the construction of the LL-37-mediated rosacea model was successful. In response to intervention with different doses of Pae (50, 100, or 150 mg/kg), LL37-mediated erythema and telangiectasis were considerably mitigated. Moreover, the application of 150 mg/kg of Pae proved to be the most effective, followed by 100 mg/kg of Pae; the 50 mg/kg paeonol group achieved minimal anti-inflammatory efficacy (**Figure 3b**). Compared to those in the model group, the average redness area and score were significantly lower in the Pae (150 mg/kg) group (0.35 ± 0.23 mm^2^ < 11.97 ± 3.73 mm^2^ and 1.00 ± 0.38 < 2.63 ± 0.59, p < 0.05 for both). These issues did not show significant improvements in the Pae (50, 100 mg/kg) groups (6.19 ± 2.38 mm^2^ < 11.97 ± 3.73 mm^2^ and 2.43 ± 0.57 < 2.63 ± 0.59, 5.16 ± 2.02 mm^2^ < 11.97 ± 3.73 mm^2^ and 2.00 ± 0.57 < 2.63 ± 0.59, all p > 0.05) (**Figures 3c, d**). However, compared to the model group, the 100 mg/kg Pae group presented greater effects (5.16 ± 2.02 mm^2^ < 6.19 ± 2.38 mm^2^ and 2.00 ± 0.57 < 2.43 ± 0.57 mm^2^) in the context of the mean redness area and score (**Figures 3c, d**). These findings collectively suggested that Pae plays a role in alleviating skin inflammation in the LL-37-mediated rosacea model mice.

### 3.3 Pae can decrease inflammatory cell infiltration in LL-37-mediated rosacea mice

Histologically, thickened epidermis, glandular hyperplasia, capillary proliferation, inflammatory cell infiltration, and destruction of muscle structure were observed locally in the model group, which were less prominent in the 150 mg/kg Pae group. Compared to those in the model group, the pathological changes described earlier were also observed in the 50 and 100 mg/kg Pae groups, but this situation improved as the concentration of the drug increased (**Figure 4a**). Inflammatory cell infiltration was statistically analyzed via hematoxylin and eosin (H&E) staining across all groups. The results revealed that the model and control groups were significantly different, indicating that the establishment of the rosacea model was successful (**Figure 4a**). However, the performance of the 150 mg/kg Pae group was not significantly different from that of the control group (p > 0.05), which indicated that inflammation levels in this group were effectively reduced. The 100 mg/kg and 50 mg/kg Pae groups showed significant differences relative to the control group (p < 0.01). However, 100 mg/kg Pae had a greater inhibitory effect on inflammatory cell infiltration than 50 mg/kg Pae (105.57 ± 2.69 < 165.43 ± 5.11). Collectively, these histological findings indicate that Pae effectively alleviates pathological inflammatory progression in LL-37-induced rosacea-like mouse skin.

**Figure 4:**
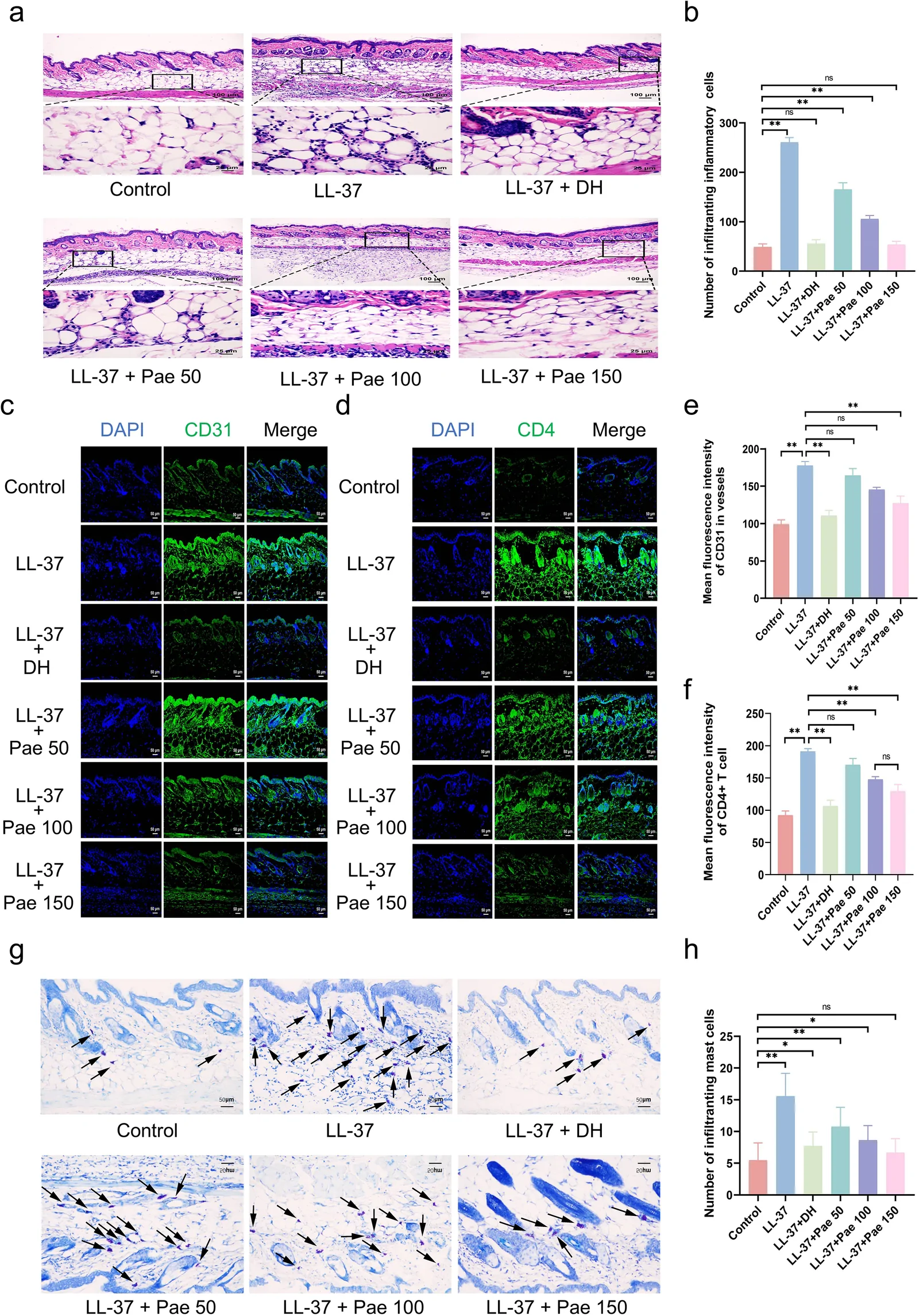
Pae can treat the pathological findings of LL-37-mediated rosacea in mice. **(a)** H&E images of rosacea-like lesions in various groups of mice are shown in the upper row. Scale bars: 100 μm and 25 μm in the upper and lower rows, respectively. **(b)** Inflammatory cell infiltration analysis of HE-stained skin tissues from LL-37-induced rosacea mice (n = 7). **(c, e)** Typical fluorescence images and the MFI for CD31 (a marker of blood vessels) expression (green) in all groups are illustrated. Blue – DAPI. Scale bars: 50 μm. The fluorescence level of CD31 in the dorsal skin lesions of the mice (n = 7). **(d, f)** Typical fluorescence images and the MFI for the CD4^+^ T-cell level (green) of all groups are illustrated. Blue-DAPI. Scale bars: 50 μm. Fluorescence level analysis of CD4^+^ T cells in the dorsal skin lesions of the mice (n = 7). **(g)** Pae decreased LL-37-mediated mast cell infiltration in rosacea mice. Mast cells infiltrated the dermis, and the black arrows indicate mast cells. Scale bars: 50 μm. **(h)** Mast cell infiltration in TB-stained skin tissues from LL-37-induced rosacea mice is shown (n = 7). The data are presented as the mean ± standard error; *p < 0.05 and **p < 0.01.

### 3.4 Pae can reduce cutaneous immune cell infiltration and angiogenesis in LL-37-induced rosacea-like mice

Skin burning and flushing are the main clinical features of rosacea. Histopathological characteristics indicate dysfunction of the cutaneous vasculature (e.g., dilation of blood vessels and angiogenesis). Thus, the angiogenic marker CD31 in the skin was used to evaluate the efficacy of Pae on angiogenesis (44, 45).

Immunofluorescence staining revealed that CD31 was significantly upregulated in the skin lesions of the LL-37-mediated rosacea model mice (p < 0.01) (**Figures 4c, e**). Compared to that in the LL-37-mediated rosacea model mice, Pae administration (50 or 100 mg/kg) led to similar CD31 levels within skin tissues in both groups, but the expression of CD31 in the 50 mg/kg Pae group was the most similar to that in the control group (164.44 ± 4.19 > 145.40 ± 1.43). In contrast, the expression of CD31 in the 150 mg/kg Pae group decreased significantly (p< 0.01). These findings indicated that a high dose of Pae effectively inhibited angiogenesis in the LL-37-mediated rosacea model mice. CD4^+^ T cells have a key effect on immune cell infiltration in rosacea (46, 47). To investigate how Pae affects the adaptive immune system, IF was conducted to examine CD4^+^ T cells in LL-37-induced rosacea mice. The expression of CD4^+^ T-cells was significantly greater in the LL37-mediated rosacea model mice than in the mice in the control group (p < 0.01) (**Figures 4d, f**). No significant difference was found in the expression of CD4^+^ T-cells between the 50 mg/kg Pae group and the LL-37-induced rosacea model group (p > 0.05). However, CD4^+^ T-cell expression was significantly lower in the 100 and 150 mg/kg Pae groups than in the LL37-induced rosacea model group (p < 0.01 for both). These results indicate that medium and high doses of Pae can effectively inhibit the infiltration of CD4^+^ T-cells. Mast cells are the main sources of LL-37 and kallikrein 5 (KLK5), the primary serine proteases responsible for the cleavage of cathelicidin into its active form. Therefore, mast cells are regarded as important participants in the pathogenesis of rosacea (48). Mast cells were stained with toluidine blue (TB), which revealed a prominent and large blue mass within the LL-37-mediated rosacea mouse skin dermis (p < 0.01); this mass decreased in size considerably after 150 mg/kg Pae was administered (p > 0.05) (**Figures 4g, h**). Although the number of mast cells in the Pae (50 and 100 mg/kg) groups increased relative to that in the control group (p > 0.05), Pae was still effective at alleviating LL-37-induced rosacea in mice, with Pae (100 mg/kg) showing greater effectiveness (10.75 ± 0.68 > 8.60 ± 0.52). To summarize, the findings of this study revealed that Pae can impede cutaneous immune cell infiltration and angiogenesis, thereby reducing LL-37-mediated rosacea-like inflammation.

### 3.5 Pae can reduce the level of rosacea-related inflammation cytokines/chemokines

Based on the results of the bioinformatics analysis involving Pae and rosacea, we confirmed the relevant cytokines/chemokines, including IL-1β, CCL2, and CXCL8, through ELISA and RT-qPCR. The above three factors were key targets for Pae improvement in mice with LL-37-mediated rosacea (**Figures 5a–e**). These results revealed that the levels of these genes were considerably different among LL-37-mediated rosacea model mice and that their expression was effectively suppressed after Pae administration. The results of the RT-qPCR assays revealed that the expression of the IL-1β, CCL2, and CXCL8 mRNAs was significantly lower in the Pae (100 and 150 mg/kg) groups than in the LL-37-induced rosacea group (p < 0.05). Specifically, the level of CCL2 mRNA was also significantly lower in the mice treated with 50 mg/kg Pae than in LL-37-induced rosacea mice (p < 0.01), indicating that Pae had the strongest effect on CCL2. The ELISA results showed that the expression of serum IL-1β was significantly lower in the Pae (100 and 150 mg/kg) groups than in the control groups (p > 0.05). A similar decrease in the expression of CCL2 was observed in Pae (100 and 150 mg/kg)-treated mice compared with the LL-37-mediated rosacea model mice (p < 0.05). These findings indicate that Pae can reduce the level of expression of potential candidate molecules associated with rosacea pathogenesis.

**Figure 5:**
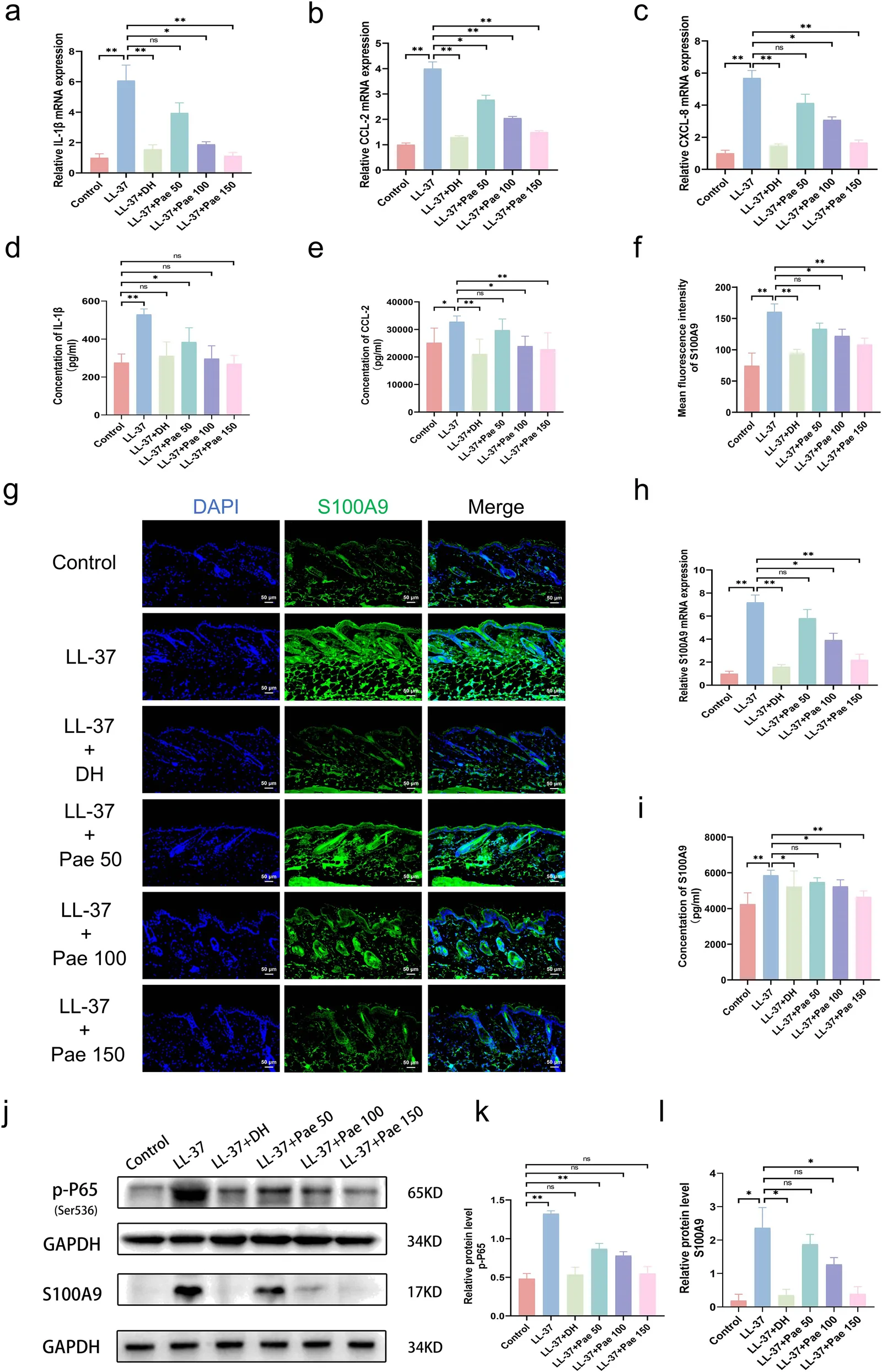
Pae reduces rosacea-related cytokine/chemokine levels, S100A9 levels, and S100A9-NF-κB pathway activation in LL-37-mediated rosacea model mice. **(a–c)** The levels of IL-1β, CCL2, and CXCL8 mRNAs in the dorsal skin lesions of the mice were determined by RT-qPCR. **(d, e)** ELISA of IL-1β and CCL2 levels in the serum. **(f)** Fluorescence level analysis of S100A9 in the dorsal skin lesions of the mice (n = 7). **(g)** Representative fluorescence images and MFI of S100A9 expression (green) in all groups are illustrated. Blue – DAPI. Scale bars: 50 μm. **(h)** The mRNA expression levels of S100A9 in the dorsal skin lesions of the mice were determined by RT-qPCR (n = 7). **(i)** ELISA of S100A9 levels in the serum (n = 7). **(j–l)** The levels of p-P65 (Ser536) and S100A9 in LL-37-induced rosacea mice from the six groups were analyzed via Western blotting, with GAPDH used as a reference (n = 4). The data are presented as the mean ± standard error; *p < 0.05 and **p < 0.01.

### 3.6 Pae can inhibit the upregulation of S100A9 in LL-37-induced rosacea mice

Similar to the findings of another study (37), we found that S100A9 was highly expressed in the skin lesions of the LL-37-mediated rosacea model mice. S100A9 was significantly upregulated in the LL-37-mediated rosacea model mice compared with the control mice (all p < 0.01, as indicated by the IF, RT-qPCR, and ELISA results) (**Figures 5f–i**). Intervention with Pae (50 mg/kg) did not significantly downregulate S100A9 (P > 0.05), as indicated by the IF, RT-qPCR, and ELISA results. However, intervention with Pae (100 and 150 mg/kg) significantly decreased the expression of S100A9 compared to that in the model group (p < 0.05); moreover, 150 mg/kg Pae was more effective than 100 mg/kg Pae in reducing the expression levels of S100A9 (122.07 ± 2.42 > 108.41 ± 2.26 in IF staining, 3.92 ± 0.22 > 2.20 ± 0.18 in RT-qPCR, 5238.08 ± 150.17 > 4653.91 ± 168.36 in ELISA). These results indicated that Pae inhibited the upregulation of S100A9 in the LL-37-mediated rosacea model mice.

### 3.7 Pae inhibited the activation of the S100A9 NF-κB pathway in LL-37-mediated rosacea model mice

The results of the Western blotting analysis suggested that the phosphorylation of p65 (a main NF-κB subunit, p-P65) increased in the LL-37-mediated rosacea model mice (p < 0.01). Pae (100 and 150 mg/kg) strongly inhibited the hyperphosphorylation of P65 (p > 0.05), and 150 mg/kg Pae was more effective than 100 mg/kg Pae (0.78 ± 0.05 > 0.55 ± 0.09) (**Figures 5j, k**). Similarly, the results of the Western blotting analysis revealed that the expression of S100A9 also increased among LL-37-induced rosacea mice (p < 0.05) (**Figures 5j, l**). Compared to the rosacea model group, Pae (150 mg/kg) significantly suppressed the expression of S100A9 (p < 0.05). Pae (50, 100 mg/kg) did not significantly suppress the expression of S100A9, and 100 mg/kg Pae still had greater effects than 50 mg/kg Pae (1.27 ± 0.20 > 0.39 ± 0.21). Combined with our analyses of paeonol-associated changes in S100A9 expression by IF, ELISA, and RT-qPCR (**Figures 5f–i**), as well as published evidence regarding the S100A9-NF-κB pathway from a previous study (37), these findings reveal a correlation between paeonol treatment and suppressed activation of the S100A9-NF-κB pathway in LL-37-mediated rosacea-like mice.

## 4 Discussion

Rosacea is a complex inflammatory disorder. It is considered to be multifactorial, but no cure or adequate control is available for rosacea. Immune dysfunction and neuroinflammation may play a role. Rosacea is mainly treated using topical and oral medications, light devices, or appropriate skin care and lifestyle management. However, most medications have several adverse reactions, and patients often stop treatment before giving sufficient time for good effects to manifest (49, 50).

Pae is an herbal phenolic compound isolated from the root bark of the traditional Chinese medicine (TCM) Moutan Cortex. Ruo et al. (51) identified 260 studies on TCM for treating rosacea for conducting a systematic review and meta-analysis; they concluded that TCM combined with standard biomedical therapy leads to better therapeutic outcomes. Iulia et al. (52) summarized the latest advances in managing rosacea via natural compounds and concluded that the benefits derived from diverse natural compounds are likely responsible for alleviating the clinical symptoms of the disease. Additionally, Pae has cosmetic effects, such as skin brightening, anti-aging, and antioxidant effects (53, 54). Rao et al. (55)developed a stable, self-emulsifying composite microemulsion (PHS-ME) co-loaded with paeonol, magnolol, and white willow bark extract, which exerts a topical anti-acne effect through their synergistic anti-inflammatory and antibacterial properties. Recent research indicates that Pae not only has anti-inflammatory effects and anti-proliferative effects but also anti-angiogenic effects (56–59). Pae can mitigate skin injury, decrease inflammatory responses among patients who develop different dermatological disorders, and suppress anomalous proliferation of skin tissue, and many researchers have developed novel formulations of Pae (23, 30, 60, 61). Pae exhibits favorable safety and tolerability in clinical practice, with rare and mild adverse-event reports. In a clinical trial investigating combination therapy of paeonol and apocynin for symptomatic knee osteoarthritis, adverse events observed were graded as mild-to-moderate. No clinically meaningful alterations were detected in clinical biochemistry, hematological indices, or urine dipstick results (62). In addition, an *in-vivo* study on rhubarb-moutan decoction for colitis demonstrated that paeonol treatment did not induce obvious abnormalities in general condition or major organs in mice (63). However, no reports on the effects of Pae on rosacea have been published. This study provides preliminary evidence for the role of Pae in alleviating LL37-induced rosacea in mouse models, and explores its potential underlying preventive mechanisms. Many researchers have reviewed the scientific literature on the biological properties of Pae in relation to skin disease and considered their potential as bioactive agents, drugs, or cosmeceuticals for treating dermatological disorders.

The S100 protein family was discovered in 1965. S100A9 belongs to the S100 subfamily of myeloid-related proteins, which have important effects on inflammation. The level of the S100A9 protein is closely related to skin disease (36). Several researchers have reported a significant increase in S100A9 in various skin diseases, including psoriasis, atopic dermatitis, rashes, and chronic non-healing wounds (64–71). However, studies on the effects of S100A9 on rosacea are rare. Our study provides a basis for subsequent studies on the mechanism underlying the effects of Pae on S100A9. Zheming et al. reported that CXCL8, IL-1β, and S100A9 were considerably upregulated in patients and mice with psoriasis and acute myocardial infarction (AMI). These findings indicate that CXCL8, IL-1β, and S100A9 can be common molecular markers and candidate therapeutic targets that link psoriasis to AMI. Our study also revealed that CXCL8, IL-1β, and S100A9 levels increased considerably in LL37-induced rosacea model mice, and Pae intervention decreased their levels. Accordingly, Pae may act as a therapeutic agent for rosacea, psoriasis, and AMI through these common molecular features and potential therapeutic targets (CXCL8, IL-1β, and S100A9). Additionally, these findings need to be verified by conducting relevant experiments. Boyi et al. (39) revealed the candidate mechanisms by which S100A9 exacerbates rosacea-related inflammation through skin transcriptome analysis and emphasized that S100A9 probably promotes the production of IL-17 to exacerbate rosacea-related inflammation, which is consistent with the results of the KEGG enrichment analysis. However, we did not experimentally validate whether paeonol ameliorates rosacea-like lesions via the S100A9-IL-17 or TLR4/MyD88-dependent cascades; this represents another area for future investigation.

Mast cells, macrophages, and CD4^+^ T cells account for most immune cells activated during rosacea (72). CD4^+^ T cells (especially Th1 and Th17 cells) were suggested to be involved in the pathogenesis of rosacea (47). However, whether Pae can inhibit Th1/Th17 polarization needs to be further investigated. Mast cells are key players in neuroimmune crosstalk, especially within the skin (73). Mast cells exhibit high activity within rosacea skin lesions and produce excessive pro-angiogenic factors, such as tryptase, histamine, fibroblast growth factor (FGF), and vascular endothelial growth factor (VEGF) (74). Our results showed that paeonol treatment is accompanied by reduced mast-cell infiltration and suppressed angiogenesis in rosacea-like mouse skin, pointing to potential links between mast cell activity and vascular abnormalities. Overall, Pae shows tremendous potential as a candidate drug for the prevention of rosacea.

It is worth noting that Pae and doxycycline hyclate displayed mixed therapeutic performances in our mouse model. Doxycycline showed better outcomes in certain inflammatory cell infiltration related parameters, whereas paeonol exerted stronger inhibitory effects on S100A9-NF-κB activation and angiogenesis. The detailed molecular mechanisms underlying these differences warrant further investigation. Pae cannot substitute for doxycycline at present and may represent a candidate for alternative or combinatorial intervention for rosacea related inflammation.

This study has also several inherent limitations. First, rosacea is a chronic relapsing skin disease, while our LL-37-induced rosacea-like mouse model is a short-term 48 h acute inflammation model, a long term efficacy, safety, or toxicity remain unproven and require future long-term studies. Second, the hypothesis that paeonol exerts its biological function via the S100A9-NF-κB signaling pathway is only supported by correlative evidence, and has not yet obtained *in vitro* functional validation. Meanwhile, PPAR-γ-related mechanisms were not characterized within the scope of the present study. All the above-mentioned points will serve as key research priorities for future experimental investigations.

## 5 Conclusion

For the first time, our findings demonstrate that paeonol markedly mitigates phenotypic manifestations of LL-37-induced rosacea-like skin inflammation in mice. Its anti-inflammatory and anti-angiogenic activities are correlated with suppressed S100A9-NF-κB signaling, although causal relationships remain to be further validated. This study suggests that paeonol may serve as a promising candidate agent for rosacea intervention.

## Data Availability

The original contributions presented in the study are included in the article/supplementary material. Further inquiries can be directed to the corresponding authors.
